# Effects of Zinc and Organic Fertilizer Amendments, Applied Individually or in Combination, on Cadmium Uptake by Wheat Grown in Alkaline Soil

**DOI:** 10.3390/plants14162525

**Published:** 2025-08-13

**Authors:** Jiang Liu, Lingxuan Kong, Yanan Wan, Qi Wang, Zhong Zhuang, Huafen Li

**Affiliations:** State Key Laboratory of Nutrient Use and Management, Beijing Key Laboratory of Farmland Soil Pollution Prevention and Remediation, College of Resources and Environmental Sciences, China Agricultural University, Beijing 100193, China

**Keywords:** wheat, cadmium, alkaline soil, zinc, organic fertilizer

## Abstract

Zinc (Zn) and organic fertilizer (OF) play a dual role in both promoting plant growth and modulating cadmium (Cd) uptake. However, the individual and combined effects of soil-applied Zn and OF on Cd accumulation in wheat remain insufficiently understood, with reported outcomes varying from inhibition to promotion of Cd uptake. Therefore, this study systematically investigated the effect of Zn, organic fertilizer, and their combined treatment on the uptake dynamics of Cd and Zn in wheat plants across different growth stages. The pot culture experiments demonstrated that applying 20 mg/kg ZnSO_4_ alone significantly reduced grain Cd content by 22.3% at the mature stage. Increasing the Zn dose to 40 mg/kg further enhanced the reduction, lowering Cd accumulation by 38.9% and decreasing Cd levels from 0.23 to 0.14 mg/kg. The application of 1% OF in alkaline soil enhanced soil Cd availability but did not significantly affect Cd accumulation in various wheat organs. The combination of Zn and organic fertilizer resulted in a relatively modest grain Cd reduction of 8.4–23.0%. Generally, Zn application alone was more effective in reducing Cd accumulation in wheat, while organic fertilizer may require careful use due to its Cd-mobilizing effect in alkaline soil. The combination of Zn and organic fertilizer showed limited benefits for Cd mitigation.

## 1. Introduction

A global map of heavy metal distribution in soils, recently published in Science, has once again brought attention to the pressing issue of soil heavy metal contamination [1]. The latest studies have confirmed that cadmium (Cd) remains the most significant soil pollutant, particularly in major agricultural regions. Additionally, a recent meta-analysis [2] reported that the proportion of farmland soils in China heavily or severely contaminated with Cd increased from 17.7% during 2000–2010 to 34.5% in 2011–2021, primarily in major crop-producing provinces such as Henan and Jiangsu. From 2000 to 2021, 25.8% of wheat grain samples were found to exceed the national Cd safety standard. Notably, from a spatial perspective, most of these samples were also observed in major crop-producing provinces, likely due to intensive industrial activities. In addition, grain-Cd contents in provinces like Yunnan, Gansu and Sichuan also exceeded the national Cd safety standard. This spatial distribution would pose a serious threat to wheat yield and food safety in China [3,4]. Therefore, the development of effective strategies to mitigate both soil cadmium contamination and its subsequent accumulation in agricultural crops has become an urgent priority.

Soil passivation technology, particularly the application of alkaline substances, demonstrates significant effectiveness in reducing the bioavailability and mobility of Cd primarily by increasing soil pH [5]. However, unlike acidic soils, alkaline soils have higher pH levels, which promote the adsorption of Cd by soil particles or the formation of compounds such as [Cd(OH)]^+^ and Cd(OH)_2_ [6]. As a result, in this case, the adsorption and formation process led to a marked decrease in Cd bioavailability and mobility [7]. Therefore, the alkaline amendments generally exhibit limited effects in alkaline soil conditions. Additionally, agronomic regulation measures, such as fertilizer management, are widely used for the remediation of heavy metal-contaminated soil due to their advantages in promoting crop growth, low cost, simple operation, and environmental friendliness.

The application of organic fertilizers introduces substantial quantities of humic substances into soil systems [8]. These complex organic molecules, containing abundant carboxyl (-COOH) and phenolic (-OH) functional groups, exhibit strong metal-chelating capabilities through the formation of coordination complexes with various metal ions. This complexation mechanism profoundly influences the speciation, mobility, and bioavailability of metals in soil environments [9]. Several studies have documented the effectiveness of farmyard manure in mitigating cadmium accumulation in crops. For example, Li et al. [10] and Wan et al. [11] demonstrated that farmyard manure application significantly reduced Cd accumulation in rice plants, with the reduction efficacy positively correlated with both application rates and duration of use. Similarly, Grüter et al. [12] reported that long-term application of quality-assured farmyard manure effectively decreased cadmium content in wheat grains. However, several studies have demonstrated that the application of organic fertilizers does not significantly influence Cd availability or its accumulation in wheat crops [13,14,15]. The remediation efficacy of organic fertilizers may vary depending on soil properties and application duration. Therefore, we aim to investigate the mitigation effectiveness of organic fertilizer amendments for cadmium contamination in alkaline soils across different growth stages.

Research has shown that zinc (Zn(II)) can help maintain cell membrane integrity under Cd stress and enhance the activity of superoxide dismutase, an enzyme participating in the detoxification of reactive oxygen species [16,17]. This helps alleviate Cd-induced toxicity and supports normal plant growth. In addition, due to their similar physicochemical properties, Zn and Cd exhibit interactive effects in soil–plant systems. During the uptake and translocation processes in plants, Zn may compete with Cd for binding sites on transport proteins, potentially influencing the bioavailability and accumulation of these metals [18].

Furthermore, the molecular mechanisms underlying Zn-mediated Cd tolerance in plants are also associated with an enhanced antioxidant system, improved photosynthetic efficiency, altered intracellular Cd distribution, and regulation of gene expression related to heavy metal transporters [19]. Numerous studies have demonstrated that Zn suppresses Cd uptake in plants. For example, Li et al. reported that soil-applied ZnSO_4_ could affect Cd content in various parts of the wheat plant by regulating the expression of transporter proteins [20]. Other research indicated Zn could reduce Cd accumulation in wheat by enhancing antioxidant enzyme activity, downregulating Cd influx transporters such as TaHMA2, TaLCT1, and ZIP family proteins, and upregulating Cd efflux transporters [21,22]. However, an earlier study found that in neutral and alkaline soil, Zn application did not reduce Cd levels in wheat roots or grains; instead, it promoted Cd translocation from roots to shoots, even at high Zn concentrations [23]. Different research results may be related to the amount of Zn added, soil properties, and plant species.

Research has demonstrated that organic fertilizer can significantly improve soil Zn availability [24,25]. Therefore, a strategic combination of organic fertilizer with Zn supplementation may offer superior potential for mitigating Cd accumulation compared to the application of either treatment independently. To address these research gaps, this study employed a pot experiment to examine the effects of Zn and organic fertilizer application on Cd accumulation at different wheat growth stages. Specifically, we aimed to: (1) investigate the temporal distribution of Cd and Zn in different wheat organs throughout the growth period, while assessing their potential variations in root uptake capacity; and (2) evaluate the variations of Cd and Zn in the rhizosphere soil following individual and combined applications of Zn and organic fertilizer. These findings aim to provide a theoretical basis for further exploration of the underlying mechanisms governing Cd and Zn transport and accumulation in wheat plants.

## 2. Materials and Methods

### 2.1. Materials

The experimental soil was collected from Cd-contaminated farmland in Henan Province, China. The soil was air-dried and passed through a 2 mm sieve prior to use. The wheat (*Triticum aestivum* L.) variety used in this study was Ningchun 4, a commonly cultivated variety in northern wheat-growing regions of China. The organic fertilizer was provided by Henan Runbosheng Environmental Technology Co., Ltd., China (Zhengzhou, China), and is a commonly used type in the region, primarily generated through composting of swine manure. The basic physical and chemical properties of the soil and organic fertilizer (OF) are presented in Table 1.

### 2.2. Pot Experiment

The wheat pot experiment was conducted from March to June 2023 in a greenhouse at China Agricultural University. Six treatments were established: a Control, OF (1%), L-Zn (20 mg/kg, as ZnSO_4_), H-Zn (40 mg/kg), and a combination of OF and Zn (OF-L-Zn, OF-H-Zn). Each treatment has three replications. Before the experiment, 5 kg of soil was thoroughly mixed with the designated amounts of OF, Zn or their combination in plastic pots. Base fertilizers were then added at rates of 0.43 g/kg urea and 0.29 g/kg potassium dihydrogen phosphate. The treated soil was left to stabilize for one week before planting. Plump wheat seeds were selected using the flotation method. Wheat seeds were soaked overnight in 10% hydrogen peroxide, followed by thorough rinsing. The cleaned seeds were then placed on a sieve and kept in the dark until they turned white. Once germinated, the seeds were evenly sown in the pots, and water was added to moisten the soil. Throughout the experiment, soil moisture was set at approximately 20%. An additional 0.43 g/kg of urea was applied at the tillering stage to support wheat growth.

Wheat and soil samples were collected at four key growth stages, namely jointing, heading, filling, and maturing. The harvested wheat plants were rinsed with tap water followed by deionized water, and then separated into root, stem, leaf, husk, and grain. These plant parts were oven-heated at 105 °C for 30 min, then dried at 70 °C until reaching a constant weight. The dried samples were ground and stored for further analysis. The collected soil samples were air-dried and passed through a 2 mm sieve for later analysis.

### 2.3. Cd and Zn Concentration Analyses

Soil-available Cd and Zn were determined using the diethylenetriaminepentaacetic acid (DTPA)-trolamine-calcium chloride extraction method, following the Chinese national environmental protection standard (HJ 803-2016) [26]. Soil and extractant were mixed at a solid-to-liquid ratio of 1:2, and the mixture was shaken at room temperature for 1 h. After shaking, the samples were centrifuged at 4000 rpm for 10 min. The resulting supernatant was filtered through a 0.45 μm membrane filter. Cd and Zn concentrations in the filtrate were measured using inductively coupled plasma mass spectrometry (ICP-MS, NexION 1000G).

For plant samples, 0.3000 g of finely ground wheat tissue was accurately weighed into a 50 mL glass digestion tube. Samples were soaked overnight in 10 mL of concentrated nitric acid (Guaranteed Reagent) and digested using an open tube electric digestion furnace (Thermo Fisher, Waltham, MA, USA). The digestion program involved heating at 120 °C for 30 min, increasing to 150 °C for 3 h, and finally raising to 180 °C to complete digestion. When the remaining volume was approximately 1 mL, the tubes were cooled and diluted to 25 mL with ultrapure water (18.2 MΩ). The solutions were then filtered through a 0.45 μm membrane and analyzed for Cd and Zn concentrations using ICP-MS. Certified reference material GBW 10049 was used for quality control, with element recovery rates between 93% and 108%.

### 2.4. Statistical Analysis

The translocation factor (TF) was usually used to evaluate the efficiency of a plant in transporting an element from the roots to the shoots (stems, leaves, or grains). TF was calculated using Equation (1):

TFj-i = Ci/Cj(1)

where *j* represents root or stem; *i* represents stem, leaf or grain; and *C* is the Cd or Zn content of root, stem, leaf or grain. For example, TF_Cd root-stem_ represents the translocation factor of Cd from root to stem.

The potential of entry (PE) for Cd and Zn was calculated using Equation (2):

PE = log_10_(root-Cd/Zn content/soil DTPA-Cd/Zn) (2)

PE-Cd and PE-Zn refer to the potential of entry of Cd and Zn, respectively.

All experimental data represent the mean of three replicates. Analysis of variance was performed using IBM SPSS Statistics 25.0 at a significance level of 0.05, and a post-hoc test was conducted using the least significant difference method. Figures were generated using Adobe Illustrator 2015 and Origin 2025.

## 3. Result

### 3.1. Temporal Variation in Cd Contents at Different Growth Stages

**Figure 1:**
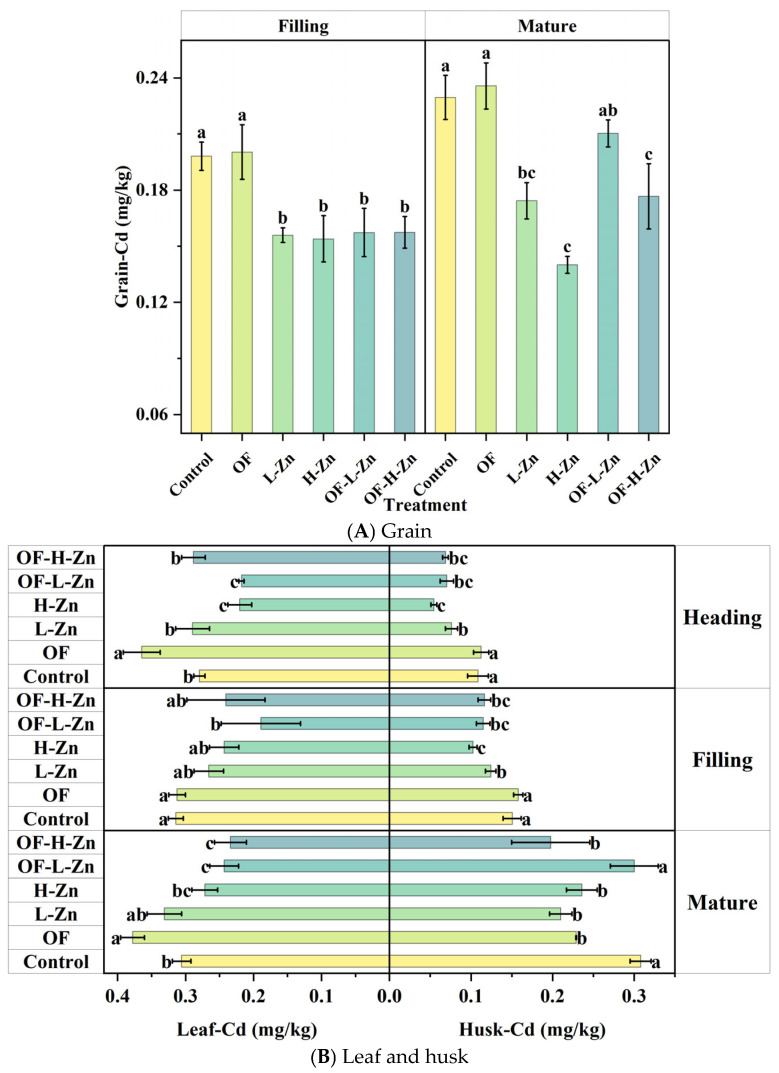

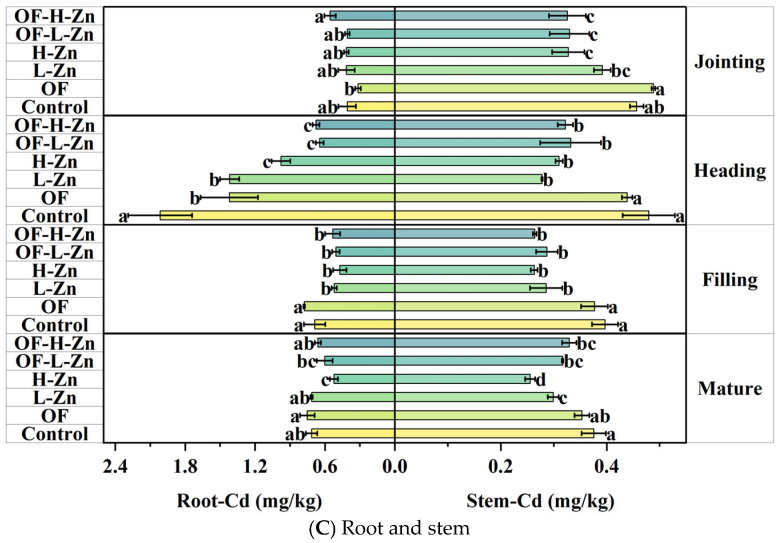
Cd content in different organs of wheat at four growth stages. Different lowercase letters represent significant differences in Cd content among different treatments for the same growth stage (*p* < 0.05).

The contents of Cd varied significantly among different growth stages and wheat organs for the Control treatment (*p* = 0.00049–0.037, Figure 1). From filling to maturity, grain-Cd and husk-Cd increased by 13.7% and 183.6%, respectively (Figure 1A,B). Leaf-Cd content remained relatively stable throughout the growth period (0.28–0.31 mg/kg), whereas stem-Cd content decreased substantially from 0.48 mg/kg to 0.38 mg/kg with the growth of wheat (Figure 1B,C). Among all treatments, the application of Zn alone exhibited the most pronounced inhibitory effect on Cd accumulation in wheat. In contrast, organic fertilizer tended to promote Cd accumulation. Notably, when Zn was combined with organic fertilizer, these treatments attenuated the suppressive effect of Zn on Cd uptake (Figure 1).

Zn application had a significant effect on decreasing Cd content (*p* < 0.05, Figure 1). Zn primarily inhibited Cd accumulation in grain, husk and stem, altering the Cd distribution pattern in a dose-dependent manner (Figure 1). The H-Zn treatment demonstrated a superior capacity for Cd reduction compared to the L-Zn treatment, achieving a 21.4% greater decrease in grain-Cd content. Furthermore, it reduced leaf-Cd by 8.5–23.9% and root-Cd by 9.2–31.1% more effectively than the L-Zn treatment. Additionally, from the heading to mature stage, compared with Control, Zn application decreased Cd content by 0.01–51.5% in root, 14.2–42.0% in stem, 14.2–29.9% in leaf, 17.5–49.8% in husk and 21.3–38.9% in grain. This indicates that Zn can effectively suppress both Cd uptake by plant roots and its subsequent translocation within plant tissues, consequently leading to a significant reduction in Cd accumulation in grain.

For organic fertilizer treatment, the temporal trends of Cd accumulation in various wheat organs were similar to those observed in the Control treatment. However, Cd levels were higher than those in the Control in most cases (Figure 1). Organic fertilizer reduced root-Cd contents by 22.8% and 29.4% respectively, at the jointing stage and heading stage (Figure 1C). The increase, however, of the leaf-Cd content (30.3%) in the heading stage (Figure 1B) may explain the decrease in root-Cd. At the filling stage, organic fertilizer had little effect on Cd content in the aboveground wheat organs. However, at the mature stage, husk-Cd content exhibited a significant decrease of 25.7%, which was accompanied by a corresponding increase of 23.6% in leaf-Cd. This shift suggests that organic fertilizer promoted Cd accumulation in the leaves.

However, when Zn was combined with organic fertilizer, these treatments mitigated the inhibitory effect of Zn on Cd accumulation (Figure 1). Compared with L-Zn alone, OF-L-Zn treatment increased Cd accumulation by 30.0% in the husk and 19.1% in the grain (Figure 1A,B). Nevertheless, the grain-Cd content in the OF-L-Zn treatment remained significantly lower than that in the organic fertilizer treatment alone (Figure 1). The combination of higher Zn with organic fertilizer at the heading stage reduced Cd accumulation in the root by 30.9% (Figure 1C), while increasing Cd content in the leaf by 30.7% (Figure 1B).

### 3.2. Temporal Variation in Zn Contents

**Figure 2:**
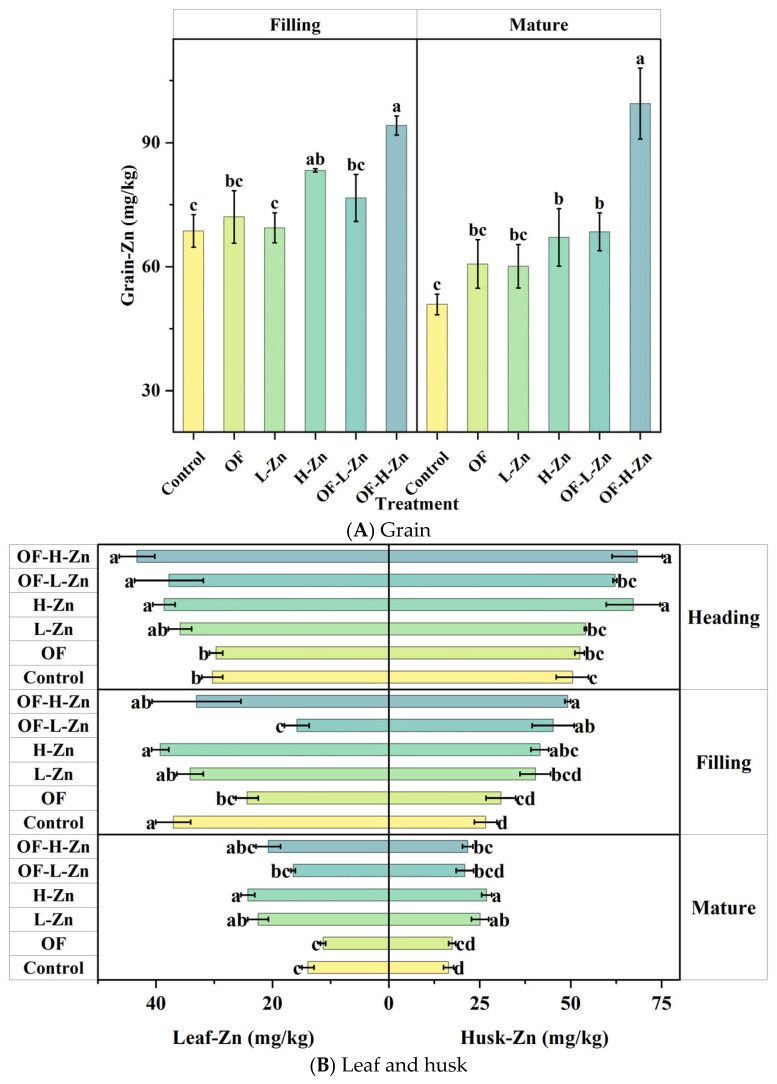

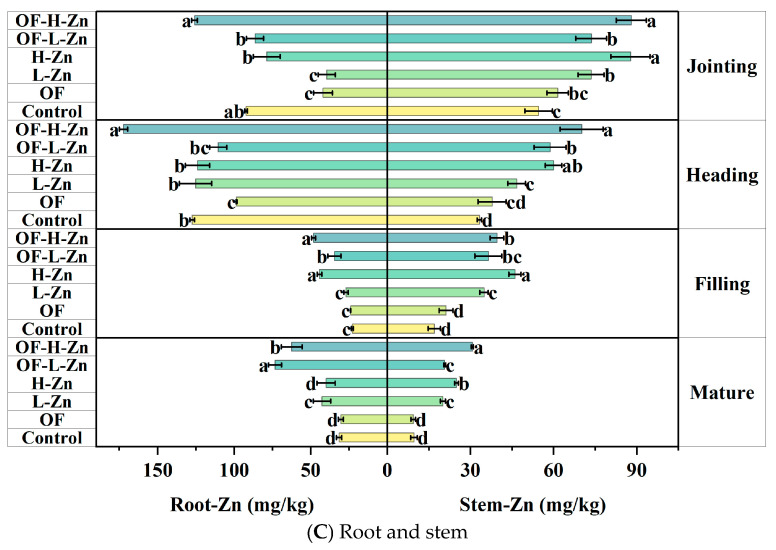
Zn content in different organs of wheat at four growth stages. Different lowercase letters represent significant differences in Zn content among different treatments for the same growth stage (*p* < 0.05).

The contents of Zn varied significantly among different wheat organs and growth stages (*p* = 0.000001–0.033, Figure 2). Zn content in grain was 1.0 to 3.8 times higher than in leaf, demonstrating substantial remobilization of Zn from vegetative tissues to developing grain during the reproductive growth phase (Figure 2A). During the growth stages from jointing to maturity, Zn contents in various wheat organs exhibited a declining trend. Specifically, leaf Zn content decreased from 30.3 mg/kg to 13.9 mg/kg, while stem Zn content showed a more pronounced reduction from 54.5 mg/kg to 9.6 mg/kg (Figure 2B,C). Interestingly, root Zn content reached its maximum at the heading stage before subsequently decreasing (Figure 2C).

The application of organic fertilizer and Zn further promoted Zn accumulation in grain (Figure 2A). Compared with Control, OF treatment increased grain-Zn content by 4.9% at the filling stage and 19.3% at the mature stage (Figure 2A). Notably, organic fertilizer significantly reduced leaf-Zn at the filling stage by 34.3% (*p* < 0.05), which may be attributed to Zn increasing in stem (25.1%, *p* > 0.05), husk (15.7%, *p* > 0.05) and grain (Figure 2). Similarly, both lower Zn and Higher Zn treatments significantly increased Zn content in the aboveground parts, with minimal differences observed in root Zn levels (Figure 2). Compared with Zn application alone, the combined application of zinc and organic fertilizer could enhance zinc accumulation in different organs.

### 3.3. Dynamics of DTPA-Extractable Cd and Zn in Rhizosphere Soil

**Figure 3:**
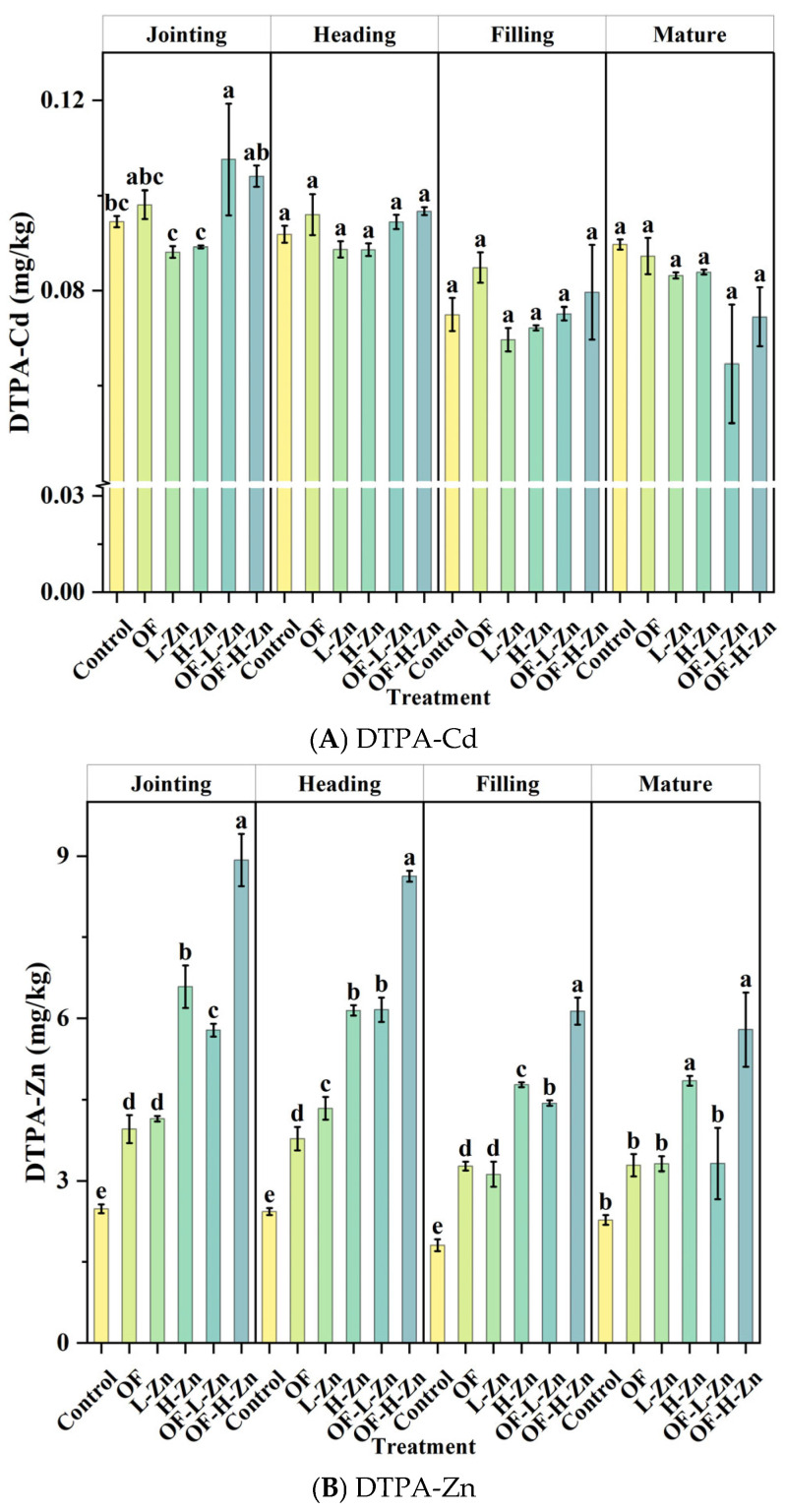
DTPA-Cd (**A**) and DTPA-Zn (**B**) content of rhizosphere soil at four growth stages. Different lowercase letters represent significant differences in Cd/Zn content among different treatments for the same growth stage (*p* < 0.05).

The content of DTPA-Cd in the rhizosphere soil ranged from 0.07 to 0.09 mg/kg across the four wheat growth stages, accounting for only 7.7–9.9% of total-Cd content, which indicates a relatively low bioavailability of Cd in the soil (Figure 3). Compared with Control, none of the treatments showed a significant effect on DTPA-Cd. In contrast, OF, L-Zn, H-Zn, OF-L-Zn, and OF-H-Zn treatments increased DTPA-Zn by 44.6–80.7%, 45.7–78.5%, 113.3–165.4%, 46.0–153.6%, and 136.2–259.7% across the four growth stages, respectively. Furthermore, in OF-L-Zn and OF-H-Zn treatments, organic fertilizer enhanced DTPA-Zn by an additional 0.2–42.1% compared to L-Zn or H-Zn alone, with the maximum content observed at the heading stage.

### 3.4. Translocation Factor of Cd and Zn

To further evaluate the effects of different treatments, we calculated the translocation factors (TF) for Cd and Zn to quantify their mobility within the plant systems (Table 2). In Control, both TF_Cd root-stem_ and TF_Zn root-stem_ decreased from jointing to the mature stage, reaching their lowest values at the heading stage. Despite this, substantial amounts of Cd and Zn were translocated from the stem to the leaf and grain. Organic fertilizer promoted greater Cd translocation to leaf and grain while inhibiting the Zn translocation. On the contrary, Zn application decreased TF_Cd root-stem_, resulting in reduced Cd transfer to grain.

At the mature stage, OF-H-Zn treatment promoted Zn translocation to the stem, which subsequently reduced both the stem-to-leaf and stem-to-grain Cd transfer factors (Table 2). This mechanism ultimately led to a significant reduction in grain-Cd content compared to OF-L-Zn treatment (*p* < 0.05, Figure 1A). Moreover, compared with OF treatment, the OF-L-Zn treatment inhibited Cd translocation from stem to leaf, while OF-H-Zn inhibited Cd translocation from stem to grain, resulting in lower grain Cd contents (Figure 1A). This supports the inhibitory role of Zn in Cd accumulation in grain. Compared with Zn treatments, organic fertilizer decreased TF_Zn root-stem_ but increased TF_Cd root-stem_ from filling to mature stage, resulting in higher grain-Cd content at the mature stage.

### 3.5. Correlation Analysis and Potential of Entry for Cd and Zn

**Figure 4:**
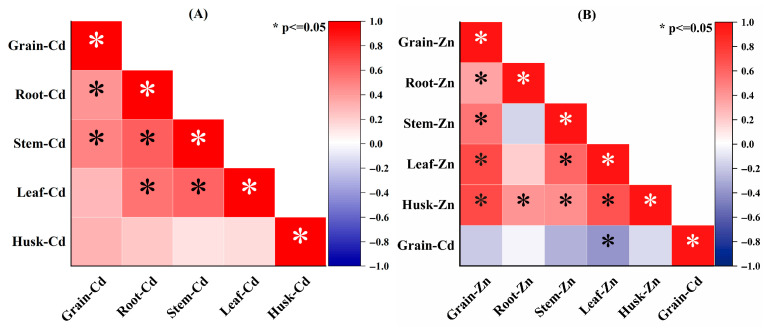
Spearman correlation coefficients among Cd (**A**) or Zn (**B**) contents of different wheat organs.

Correlation analysis revealed that grain-Cd content exhibited a positive correlation with root-Cd and stem-Cd, while showing a negative correlation with leaf-Zn (Figure 4). These findings indicate that minimizing root Cd absorption and restricting Cd translocation to the stem would directly decrease grain-Cd content. The results further demonstrated that Zn application significantly enhanced Zn accumulation in leaves while effectively reducing Cd contents in stem and root (*p* < 0.05, Figure 2B).

**Figure 5:**
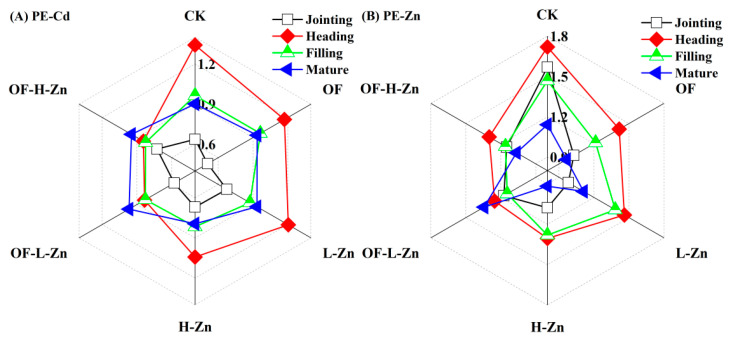
PE-Cd and PE-Zn at different growth stages of wheat.

The potential of entry (PE) was proposed in this study as a quantitative measure to evaluate the efficiency of element uptake from soil into plant roots. Results indicated that both PE-Cd and PE-Zn reached their peak levels at the heading stage, and PE-Zn decreased after the heading stage (Figure 5). Compared with Control, organic fertilizer promoted Cd uptake while simultaneously inhibiting Zn entry into the root during the filling and mature stages.

The observed reduction in PE-Cd under Zn treatment likely contributed to the decreased Cd accumulation in wheat roots. Additionally, the difference in PE-Cd between the L-Zn and H-Zn treatments may account for the variation in Cd accumulation in the root, especially at heading and mature stages. The combined application of Zn and organic fertilizer demonstrated greater efficacy in reducing PE-Cd than Zn alone before the mature stage. However, the higher TF_Cd root-stem_ and TF_Cd stem-grain_ suggest that the decrease in root-Cd was more related to the translocation rather than PE-Cd.

The decrease in PE-Zn did not lead to a corresponding reduction in root Zn content. This is mainly because the increases in DTPA-Zn at each stage were greater than the increases in root Zn content. For example, at the filling stage, root Zn contents in the treatments were 0.05–1.11 times higher than the Control, whereas the corresponding DTPA-Zn levels were 0.81–2.39 times higher. Notably, at the mature stage, the OF-L-Zn treatment exhibited a higher PE-Zn, resulting in greater Zn accumulation in the root but lower in the aboveground.

## 4. Discussion

### 4.1. Effect of Zn on Cd Accumulation in Wheat

Because Cd and Zn share the common transport pathways, primarily mediated by zinc/iron-regulated transporter-like proteins and yellow stripe 1-like proteins [27,28,29], the presence of Zn in soil can compete with Cd during plant root absorption. The results of the present study show that Zn application significantly reduced Cd content in different wheat organs, particularly at the higher application rate (Figure 1). However, in the study by Sarwar et al. [23], soil DTPA-Zn accounted for only 0.6% of total Zn, suggesting that externally applied Zn may compete with Cd for adsorption sites on soil minerals [30], thereby increasing Cd availability and its uptake by wheat. In contrast, our study found that DTPA-Zn accounted for 4.1% of total Zn, indicating that externally applied Zn had a negligible effect on DTPA-Cd across different growth stages (Figure 3A). Furthermore, compared with the H-Zn treatment, L-Zn reduced Cd uptake by decreasing PE-Cd while increasing PE-Zn, as evidenced by the lower root Cd content at the heading and filling stages (Figure 1C) and the changes in PE-Cd and PE-Zn (Figure 5). This is likely due to the shared transporter proteins between Zn and Cd, and the ability of Zn to downregulate the expression of related genes, as previously mentioned.

Since root Cd content alone cannot fully explain the reduction in grain Cd, the 24.3% increase in stem Zn at the mature stage may help clarify why the H-Zn treatment reduced grain Cd more effectively than the L-Zn treatment (Figure 1A or Figure 2C). This is likely because both Zn and Cd must be translocated into the phloem within the stem by binding to specific proteins during their movement from shoot to grain [31]. The increase in stem Zn may therefore reduce the TF_Cd stem–grain_ value at the mature stage (Table 2). Therefore, another possible explanation for Zn decreasing grain-Cd content is that induced Cd accumulation in the leaf limits its movement into the grain. More importantly, the high leaf-Zn content further promoted Zn translocation to the grain, thus restricting Cd entry by competitive inhibition. This helps explain the observed negative correlation between grain Cd and leaf Zn (Figure 4B). When comparing the L-Zn and H-Zn treatments, the lower Zn dosage appeared to reduce grain Cd levels by promoting Cd retention in the roots or leaves (Figure 1B,C) and limiting its translocation. In contrast, the higher Zn dosage tended to inhibit Cd absorption and translocation, resulting in lower Cd accumulation (Figure 1 and Figure 4). This may be due to sufficient Zn supply suppressing the root secretion of phytosiderophores, as confirmed by previous research [32,33]. Since phytosiderophores can chelate both Zn and Cd, their reduced release may consequently limit Cd uptake [34].

However, Zn can also enhance Cd bioavailability in soil by competing with Cd for adsorption sites on soil mineral surfaces [23,30]. Consequently, the net impact of Zn on Cd uptake by plants depends on the complex balance between soil characteristics and plant physiological mechanisms. The present study demonstrates that Zn application had no significant effect on Cd availability in the soil (Figure 3), but significantly influenced Cd uptake by wheat plants. Overall, based on the competitive relationship between Zn and Cd and our experimental results, Zn appears to reduce Cd accumulation in wheat grain through three key plant physiological mechanisms: (1) decreasing Cd absorption by roots, (2) inhibiting Cd translocation to the stem and grain, and (3) increasing Zn content in leaves by promoting Zn translocation from root to shoot, thereby further restricting Cd movement within the plant.

### 4.2. Effect of Organic Fertilizer on Cd Accumulation in Wheat

In this study, organic fertilizer tended to promote Cd translocation to the stem and leaf before the filling stage, and to the grain thereafter; however, it had no significant effect on root or grain Cd content after the heading stage (Table 2, Figure 1). Notably, the effectiveness of organic fertilizer observed in our study was different from the previous research. For example, Azhar et al. [35] reported that organic fertilizer could decrease DTPA-Cd content in alkaline soil. This may be because the DTPA-Cd content in their study was relatively high (~8.0 mg/kg). As a result, the application of organic fertilizer reduced DTPA-Cd levels and ultimately lowered Cd accumulation in wheat. In contrast, the DTPA-Cd content in our study was only 0.07–0.09 mg/kg. Therefore, organic fertilizer did not enhance Cd passivation in the soil (Figure 3), and consequently, no significant reduction in wheat Cd content was observed (Figure 1). In addition, long-term field studies in acidic soils found that applying organic fertilizer for 7 years reduced both soil Cd availability and grain Cd content [12]. However, short-term application (1 year) actually increased soil Cd availability and Cd uptake by wheat [36]. In our study, the organic fertilizer was applied for only 3 months, yet afforded similar results, suggesting that the duration of application may influence its effectiveness in reducing Cd accumulation. Moreover, in the study by Zhao et al. [37] and Shen et al. [38], the soils were acidic with higher Cd availability. In those cases, application of the organic fertilizer significantly increased soil pH, thereby reducing Cd availability. On the contrary, in our study, the soil was alkaline, and the application of organic fertilizer did not further increase soil pH. Combined with the already low Cd availability, this resulted in a limited effect of organic fertilizer on reducing Cd content in wheat. Therefore, additional remediation measures are needed to effectively control Cd accumulation under such conditions.

Furthermore, the decomposition of organic fertilizers in soil increases the concentration of dissolved organic carbon (DOC), which facilitates the formation of DOC–Cd complexes [39,40]. Under alkaline pH conditions, the formation of DOC-Cd complexes can reduce Cd adsorption onto soil carbonate minerals, thereby increasing Cd availability [41,42,43]. Specifically, organic fertilizer increased DTPA-Zn (Figure 3) but decreased its potential of entry (Figure 5). This phenomenon is likely attributed to the Zn inputs from fertilizer itself (Table 1) and the formation of soluble metal-organic complexes, which are generally less bioavailable to plants than inorganic Zn species [12].

In fact, compared with Zn application alone, the combined application of organic fertilizer and Zn mitigated the inhibitory effect of Zn on Cd accumulation in grain at the mature stage (*p* > 0.05, Figure 1). For OF-L-Zn treatment, this was likely due to a higher Cd translocation factor (Table 2). Additionally, although root Zn content was elevated, a lower Zn translocation factor resulted in reduced leaf Zn content (Table 2, Figure 2B), ultimately contributing to increased grain Cd content (Figure 1), as discussed in Section 4.1. As leaf Zn content was negatively correlated with grain Cd content (Figure 4), the limited Zn translocation from stem to leaf in OF-H-Zn treatment (Table 2) may explain why it has a higher grain Cd content than the H-Zn treatment alone (Figure 1). Additionally, the relatively higher leaf Zn content in the OF-H-Zn treatment (Figure 2) could explain why its grain Cd content is lower than that of the OF-L-Zn treatment. However, no significant differences were observed at the filling stage, which may be attributed to the overall lower PE-Cd values during this period (Figure 5), leading to reduced Cd accumulation in wheat tissues (Figure 1).

## 5. Conclusions

Adequate Zn application effectively suppressed Cd uptake by roots while promoting Zn accumulation in leaves, consequently reducing Cd levels in grains. However, insufficient Zn supply would limit the sustained effectiveness of this mitigation. The application of organic fertilizer in alkaline soil was observed to promote the Cd activation in the rhizosphere soil, while showing no significant effect on Cd accumulation in wheat grain. The combined application of Zn and organic fertilizer reduced the effectiveness of Zn in mitigating Cd accumulation in wheat plants. Zn application alone was more effective in decreasing Cd accumulation in wheat grain under alkaline soil conditions, while organic fertilizer may require careful use due to its Cd-mobilizing effect in alkaline soil.

## Figures and Tables

**Table 1:** Physical and chemical properties of soil and organic fertilizer.

Item	Total Cd (mg/kg)	Total Zn (mg/kg)	pH	Cation Exchange Capacity cmol/kg	Organic Matter (g/kg)	Available P (mg/kg)	Available K (mg/kg)	Alkaline Hydrolysis N (mg/kg)
Soil	0.91	82.6	8.5	19.5	11.9	20.8	187.0	52.0
OF	0.63	643.8	7.9	55.1	494.3	1.3 × 10^3^	1.5 × 10^4^	5.9 × 10^3^

**Table 2:** Translocation factors of Cd and Zn between different parts.

Treatment		Control	OF	L-Zn	H-Zn	OF-L-Zn	OF-H-Zn
TF_Cd root-stem_	Jointing	1.12 ± 0.20	1.54 ± 0.13	0.94 ± 0.04	0.78 ± 0.03	0.81 ± 0.05	0.59 ± 0.05
	Heading	0.24 ± 0.02	0.31 ± 0.06	0.20 ± 0.05	0.32 ± 0.02	0.51 ± 0.08	0.48 ± 0.04
	Filling	0.58 ± 0.03	0.48 ± 0.03	0.55 ± 0.04	0.56 ± 0.05	0.57 ± 0.02	0.49 ± 0.07
	Mature	0.53 ± 0.04	0.47 ± 0.05	0.42 ± 0.02	0.49 ± 0.02	0.53 ± 0.06	0.50 ± 0.06
TF_Zn root-stem_	Jointing	0.59 ± 0.06	1.46 ± 0.30	1.86 ± 0.42	1.12 ± 0.10	0.85 ± 0.09	0.70 ± 0.06
	Heading	0.26 ± 0.01	0.38 ± 0.06	0.37 ± 0.01	0.48 ± 0.01	0.53 ± 0.02	0.41 ± 0.04
	Filling	0.32 ± 0.07	0.40 ± 0.01	0.46 ± 0.03	0.51 ± 0.03	0.59 ± 0.08	0.45 ± 0.07
	Mature	0.31 ± 0.05	0.31 ± 0.01	0.47 ± 0.02	0.62 ± 0.10	0.28 ± 0.02	0.49 ± 0.06
TF_Cd stem-leaf_	Heading	0.58 ± 0.06	0.83 ± 0.05	1.04 ± 0.20	0.71 ± 0.06	0.66 ± 0.07	0.90 ± 0.05
	Filling	0.88 ± 0.13	0.83 ± 0.08	0.93 ± 0.10	0.93 ± 0.10	0.66 ± 0.06	0.91 ± 0.23
	Mature	0.84 ± 0.04	1.07 ± 0.12	1.11 ± 0.08	1.06 ± 0.12	0.77 ± 0.09	0.71 ± 0.01
TF_Zn stem-leaf_	Heading	0.91 ± 0.07	0.78 ± 0.11	0.77 ± 0.06	0.64 ± 0.07	0.64 ± 0.08	0.62 ± 0.11
	Filling	2.19 ± 0.46	1.15 ± 0.20	0.98 ± 0.01	0.85 ± 0.07	0.43 ± 0.06	0.84 ± 0.20
	Mature	1.45 ± 0.07	1.21 ± 0.07	1.12 ± 0.18	0.97 ± 0.03	0.79 ± 0.03	0.68 ± 0.04
TF_Cd stem-grain_	Filling	0.50 ± 0.01	0.53 ± 0.03	0.55 ± 0.07	0.59 ± 0.04	0.55 ± 0.09	0.60 ± 0.03
	Mature	0.61 ± 0.05	0.67 ± 0.01	0.58 ± 0.03	0.55 ± 0.05	0.66 ± 0.06	0.54 ± 0.05
TF_Zn stem-grain_	Filling	4.05 ± 0.67	3.40 ± 0.67	1.99 ± 0.04	1.81 ± 0.11	2.10 ± 0.14	2.38 ± 0.07
	Mature	5.29 ± 0.83	6.49 ± 0.54	2.99 ± 0.16	2.69 ± 0.35	3.31 ± 0.21	3.24 ± 0.22

## Data Availability

The data presented in this study are available on request from the corresponding author. The data are not publicly available due to privacy and ethical restrictions.
